# Biomolecular and quantum algorithms for the dominating set problem in arbitrary networks

**DOI:** 10.1038/s41598-023-30600-4

**Published:** 2023-03-14

**Authors:** Renata Wong, Weng-Long Chang, Wen-Yu Chung, Athanasios V. Vasilakos

**Affiliations:** 1 Physics Division, National Center for Theoretical Sciences, National Taiwan University, Taipei, 10617 Taiwan; 2 Department of Computer Science and Information Engineering, National Kaohsiung University of Science and Technology, Kaohsiung, 807618 Taiwan; 3 Center for AI Research (CAIR), University of Agder (UiA), Grimstad, Norway

**Keywords:** Computational biology and bioinformatics, Nanoscience and technology

## Abstract

A dominating set of a graph $G = (V, E)$ is a subset *U* of its vertices *V*, such that any vertex of *G* is either in *U*, or has a neighbor in *U*. The dominating-set problem is to find a minimum dominating set in *G*. Dominating sets are of critical importance for various types of networks/graphs, and find therefore potential applications in many fields. Particularly, in the area of communication, dominating sets are prominently used in the efficient organization of large-scale wireless *ad hoc* and sensor networks. However, the dominating set problem is also a hard optimization problem and thus currently is not efficiently solvable on classical computers. Here, we propose a biomolecular and a quantum algorithm for this problem, where the quantum algorithm provides a quadratic speedup over any classical algorithm. We show that the dominating set problem can be solved in $O(2^{n/2})$ queries by our proposed quantum algorithm, where *n* is the number of vertices in *G*. We also demonstrate that our quantum algorithm is the best known procedure to date for this problem. We confirm the correctness of our algorithm by executing it on IBM Quantum’s qasm simulator and the Brooklyn superconducting quantum device. And lastly, we show that molecular solutions obtained from solving the dominating set problem are represented in terms of a unit vector in a finite-dimensional Hilbert space.

## Introduction

For a function $H : {a| 0 \le a \le 2^n - 1} \rightarrow {b| 0 \le b \le 2^m - 1}$, the *r*-element distinctness problem is to find *r*-distinct elements $a_1, a_2, \ldots , a_r \in \{a| 0 \le a \le 2^n - 1\}$ such that $H(a_1) = H(a_2) = \cdots = H(a_r)$^1^. The *r*-element distinctness problem was later extended by Childs and Eisenberg^2^ to solve the much more general problem of finding a subset of size *r* that satisfies any given property. The dominating set problem can be regarded as the problem of finding a subset *U* of vertices of size *r* that satisfies *U* to be a minimum-size dominating set in *G*.

In 2007, Ambainis^3^ proposed an $O(2^{2n/3})$-query quantum walk algorithm for 2-element distinctness, and, more generally, an $O(2^{nr/(r+1)})$-query quantum walk algorithm for finding *r* equal numbers. The lower bound of any quantum algorithm for solving any NP-complete problem is $\Omega (2^{n/2})$, where *n* is the input size (number of bits) of that problem^4^. This implies that a quantum algorithm for solving any NP-complete problem is optimal if its upper bound is $O(2^{n/2})$.

In this work, we propose a quantum algorithm that provides a quadratic speedup over any classical algorithm. While solving NP-complete problems such as the dominating set problem classically, one often employs heuristics. Heuristics are known for finding approximate or even optimal solutions given certain restrictive assumptions about the structure of the problem. This is for instance the case with a greedy leaf removal-based procedure^5^, which can achieve optimal solution if the underlying graph is sufficiently small or sparse. Such algorithms can achieve even linear asymptotic running times, e.g. Chebolu et al.^6^, do so however for a narrow subset of the given problem, such as sparse graphs only. A logarithmic running time $O(\log d)$ can be obtained for graphs of maximum degree *d* by the greedy approximation algorithm^7^. There exist also exact classical algorithms for the problem that run in exponential time albeit slightly less than $O(n2^n)$, such as $O(1.5048^n)$ time algorithm discovered by van Rooij et al.^8^. Our claim of quadratic speedup over classical algorithms does not apply to classical heuristics. For more details the reader is advised to consult e.g.^9^. Our quantum algorithm is designed to give a quadratic speedup for any kind of graph structure without restrictions.

In this work, we also improve on the algorithm in Chang and Guo^10^ by proposing a new solution space construction and extending it to accommodate various methods of bit encoding using DNA. And lastly, we show that the proposed quantum algorithm is the best known to date.

**Figure 1:**
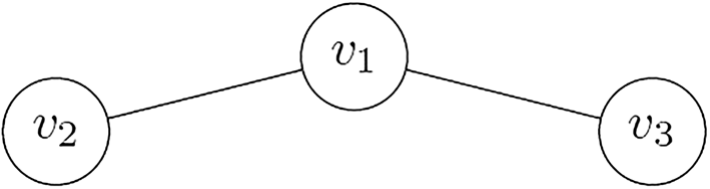
Example graph with $V = \{v_1, v_2, v_3\}$ and $E = \{(v_1, v_2), (v_1, v_3)\}$. The minimum dominating set is $\{v_1\}$.

Let a network be represented by a graph $G = (V,E)$ where *V* is the set of vertices with $|V| = n$ and *E* is the set of edges with $|E| = z$. A dominating set of *G* is a subset $U \subseteq V$ such that for all $v \in V - U$ there exists a $u \in U$ such that $(v,u) \in E$. The dominating-set problem is to find a minimum-size dominating set in *G*. It is is a NP-complete problem. Figure 1 shows a graph with $V = \{v_1, v_2, v_3\}$ and $E = \{(v_1, v_2), (v_1, v_3)\}$. The minimum dominating set for this graph is $\{v_1\}$.

Dominating sets are prominently used in the efficient organization of large-scale wireless *ad hoc* and sensor networks. Large networks usually require a certain structural organization in order for them to operate efficiently. The most prominent of such structures are based on dominating and independent sets^11^. An example of efficient allocation of resources in a network are cluster-based control structures. This type of structures allows one to view the network hierarchically, which reduces the complexity of the network. Clustering involves grouping nodes into units that are controlled by one designated node. In many approaches, these control nodes form an independent set. On the other hand, dominating sets play a crucial role in alleviating the broadcast storm problem^12^. In wireless sensor networks, dominating sets help to achieve energy conservation in sensors thereby prolonging the lifetime of such networks. An important way in energy conservation is the application of the so-called sleep-wake scheduling, where data gathering and sensing tasks are allocated to a dominating set of awake sensors while the other nodes are in a sleep mode^13^.

In general, dominating sets are crucial in efficient control, tracking, or detection of the behavior of the constituent nodes of a network^14^. The present paper focuses on the issue of dominating sets and how to find them, including the minimum dominating sets, in arbitrary graphs/networks. For a quantum algorithm solving the independent set problem in networking, the reader is referred to Chang et al.^15^

The rest of the article is organized as follows: in the next section we provide the motivation for the present manuscript and summarize our main results. Then, we introduce the relevant biomolecular and quantum operations. After that, we derive our biomolecular algorithm and our quantum algorithm for the dominating set problem and show how the quantum algorithm relates to the biomolecular algorithm. Complexity assessment and experimental validation of the quantum algorithm are provided in the subsequent sections.

## Motivation and main results

As stated in the Introduction, Bennett et al.^4^ have shown that the quantum lower bound for solving any NP-complete problem is $\Omega (2^{n/2})$, where *n* is the input size. This implies that a quantum algorithm for solving any NP-complete problem is optimal if its upper bound is $O(2^{n/2})$. The motivation for the present work was to design an optimal quantum algorithm for solving an instance of the dominating-set problem in a graph *G*.

Our main contributions in this paper are as follows:

- We show that the dominating-set problem for a graph *G* with *n* vertices and *z* edges can be solved in $O(2^{n/2})$ queries.
- We demonstrate that the proposed quantum algorithm is optimal.
- We validate the proposed algorithm for the case of a graph with three vertices and two edges by executing it on the IBM qasm simulator and the Brooklyn backend (a 65-qubit system).
- We show that molecular solutions obtained from solving the dominating set problem are represented in terms of a unit vector in a finite-dimensional Hilbert space.

## Introduction to biomolecular and quantum operations

### Biomolecular operations

In this section we introduce the biomolecular and quantum operations that are relevant for understanding our biomolecular and quantum algorithms. The biomolecular operations employed in this paper are introduced in^10,16–18^, utilized in e.g.^15,19–21^ , and briefly presented below. In the following it is assumed that tubes $X = \{x_{n}x_{n-1} \ldots x_1 | x_d \in \{0, 1\}, 1 \le d \le n\}$, and that a superscript of 0 or 1 indicates that the superscripted bit holds the value 0 or 1, respectively.

1. Given a tube *X* and a DNA strand $x_j$, the operation *Append-Tail* appends $x_j$ onto the end of every element in *X*. Formally: $Append_Tail(X, x_j) = \{x_{n}x_{n-1} \ldots x_{1}x_j\}$. This is achieved by means of denaturation and annealing.
2. Given *m* tubes $X_1, \ldots , X_m$, the *Merge* operation unifies their content, i.e., $Merge(X_1, \ldots , X_m) = X_1 \cup \ldots \cup X_m$. This is achieved by pouring the contents of the tubes into a single tube.
3. Given a tube *X*, the operation $Amplify(X, \{X_i\})$ generates a number of identical copies $X_i$ of *X* and then discards *X*. This is achieved by polymerase chain reaction.
4. Given a tube *X* and a strand $x_j$, if $x_j = 1$ (this can be indicated with $x_j^1$) then the *Extract* operation creates two new tubes $+(X, X^1_j) = \{ x_n \ldots x^1_j \ldots x_1 \}$ and $-(X, X^1_j) = \{ x_n \ldots x^0_j \ldots x_1 \}$. This is achieved by affinity chromatography.
5. The operation *Discard*(*X*) is achieved by pouring out the content of *X*.
6. Given a tube *X*, the operation *Detect*(*X*) returns a True if $X \ne \emptyset$, i.e., the tube is not empty. Otherwise, it returns a False.
7. Given a tube *X*, the bio-molecular operation *Read*(*X*) describes any element in *X*. Even if *X* includes many different elements, this operation can give an explicit description of exactly one of them.

### Quantum operations

A qubit $$|\beta \rangle =l_1 |0\rangle +l_2 |1\rangle =\begin{bmatrix}l_1&l_2 \end{bmatrix}^T$$ is defined as a linear combination of two computational basis vectors $|0\rangle$ and $|1\rangle$ of the two-dimensional complex Hilbert space, where $$|0\rangle =\begin{bmatrix}1&0 \end{bmatrix}^T$$ and $$|1\rangle =\begin{bmatrix}0&1 \end{bmatrix}^T$$, and the weighted factors $l_1$ and $l_2 \in \textbf{C}$ are the so-called probability amplitudes that satisfy $|l_1|^2 + |l_2|^2 = 1$. A collection of *n* qubits is called a quantum register of size *n*. The state of a quantum register $|\psi \rangle$ is mathematically represented by the tensor product $|\psi \rangle =\bigotimes _{d=n}^1 |\gamma _d\rangle$. The time evolution of a quantum state is modeled by unitary operators, which are often referred to as quantum gates. A quantum gate can thus be regarded as an elementary quantum-computing device that completes a fixed unitary operation on selected qubits during a fixed period of time.

A quantum algorithm is a process consisting in the following steps:

- Initialize the quantum system in a desired state $|\psi \rangle$
- Sequentially apply quantum gates to the entire system or a subsystem thereof to compute a desired problem
- Measure the resulting quantum state of the system or a subsystem thereof in order to obtain a desired outcome with a certain, specified probability

Quantum gates can be understood in analogy to classical logic gates. Some quantum gates have in fact their corresponding classical gates. However, as quantum computing is grounded in the principles of quantum mechanics, certain quantum gates, such as the Hadamard-Walsh gate, which sets a given quantum state into a superposition, do not have their classical counterparts.

For the purpose of this work, we introduce the following quantum gates:

- The NOT gate, often referred to as X gate, flips the value of a qubit from 0 to 1 and *vice versa*. It can therefore be understood as the operation of negation.
- The above mentioned Hadamard-Walsh gate, usually abbreviated as H
- The CNOT (controlled-NOT) gate is a two-qubit gate that flips the second qubit (target qubit) if and only if the first qubit (control qubit) is equal to 1.
- The Toffoli gate, often referred to as CCNOT, as an abbreviation for controlled-controlled NOT gate. This gate has two control qubits and a single target qubit. If both control qubits are in the state 1, the bit value of the target qubit will be flipped.

**Figure 2:**
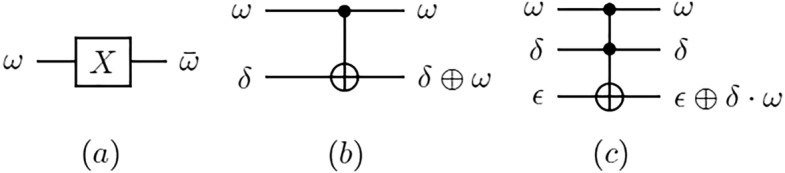
(**a**) NOT , (**b**) CNOT, (**c**) CCNOT. A bar indicates negation. A $\oplus$ indicates the exclusive logic OR operation.

Figure 2 shows the conventional graphical representation for these three gates, which often are also referred to as X, CX, and CCX.

Quantum entanglement is achieved for quantum systems of two or more qubits. The simplest example of this is a two-qubit system with a Hadamard and a CNOT gate. The matrix elements of the Hadamard gate H are $H_{1,1} = 1/\sqrt{2}, H_{1,2} = 1/\sqrt{2}, H_{2,1} = 1/\sqrt{2}$ and $H_{2,2} = -1/\sqrt{2}$. For a general input $|\beta \rangle$, it produces the following quantum state vector

$$\begin{aligned} |\phi \rangle =H|\beta \rangle =\frac{l_1+l_2}{\sqrt{2}}|0\rangle +\frac{l_1-l_2}{\sqrt{2}}|1\rangle \end{aligned}$$

It follows that $H|0\rangle =(|0\rangle +|1\rangle )/\sqrt{2}=|+\rangle$ and $H|1\rangle =(|0\rangle -|1\rangle )/\sqrt{2}=|-\rangle$. Applied to a quantum register of *n* qubits initialized to $|0\rangle$ the gate gives

$$\begin{aligned} |\delta \rangle =H^{\otimes n} |0^{\otimes n}\rangle =\frac{l}{\sqrt{2}} \sum _{i=0}^{2^n-1} |i\rangle \end{aligned}$$

For more details on quantum gates the reader is referred to^22^.

## Solving the dominating set problem

In this section, we describe, step by step, our biomolecular algorithm for the dominating set problem. After each step we show how the corresponding part can be implemented using quantum circuits.

### Generating dominating set candidate space

In the first step of the algorithm, the dominating set candidate space is created which consists of $2^n$ candidate dominating sets, where *n* is the size of the problem, i.e. the number of vertices in graph *G*. The dominating set space consists of all possible sets of vertices, which may or may not be dominating sets.

A binary string $x_n, x_{n-1}, \ldots , x_1$ of *n* bits is used to represent each possible dominating set in *G*, where $x_i \in \{0,1\}$ and $1 \le i \le n$. $x_i$ encodes the *i*-th vertex in *G*. Suppose that *U* is a dominating set in *G*. If the *i*-th vertex is in *U*, then $x_i$ is set to 1. Otherwise, it is set to 0. By doing this, all of the possible dominating sets in *G* are transformed into an ensemble of all binary numbers of *n* bits.

Given that the graph in Fig. 1 contains three vertices, a binary number of three bits in length will encode each of the $2^3$ dominating set candidates. Namely $x_3^0 x_2^0 x_1^0 (000)$, $x_3^0 x_2^0 x_1^1 (001)$ through $x_3^1 x_2^1 x_1^1 (111)$, encode the 8 candidates: $\emptyset$, $\{v_1\}$, $\{v_2\}$, $\{v_2, v_1\}$, $\{v_3\}$, $\{v_3, v_1\}$, $\{v_3, v_2\}$ and $\{v_3, v_2, v_1\}$. Not every of those candidates however corresponds to a legal dominating-set.

**Figure 3:**
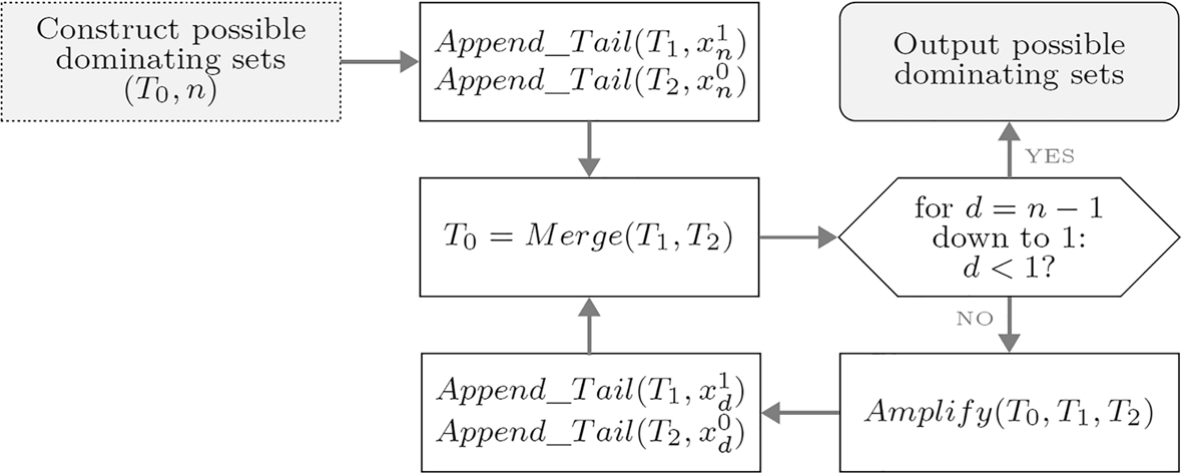
Biomolecular procedure for constructing the dominating set candidate space.

A biomolecular procedure that constructs such a space is shown in Fig. 3. A lab setting for this procedure involves three empty tubes $T_0$, $T_1$ and $T_2$. Operation *Append_Tail* appends a segment encoding bit $x_n$ with value either 1 or 0 onto the end of all strands in tubes $T_1$ and $T_2$, respectively. Operation *Merge* then combines the contents of $T_1$ and $T_2$ into a new tube $T_0$. Next, in each step for $d = n - 1$ down to 1, two copies $T_1$ and $T_2$ of $T_0$ are generated, and $T_0 = \emptyset$. Then, upon each execution of amplification, a strand encoding $x^1_d$ ($x^0_d$) is appended to the end of all strands in $T_1$ ($T_2$). These two tubes are then emptied after being merged into $T_0$. The procedure terminates with $T_0 = \{x_n \ldots x_2 x_1| x_d \in \{0,1\}, 1 \le d \le n$.

For the quantum case, let the initial quantum state vector be $|\theta _0\rangle =|1\rangle \otimes (\bigotimes _{i=n}^1 |x_i^0\rangle )$. The solution space with $2^n$ elements is generated by applying the Hadamard-Welsh gate to the *n* qubits in the state $\bigotimes _{i=n}^1 |x_i^0\rangle$, as shown in (1). The first qubit $|1\rangle$ will be used for amplitude amplification in later stages of the algorithm. The Hadamard gate acting on this qubit gives $H|ket{1}=|-\rangle$, which is used to mark the answers among $2^n$ states in Grover’s oracle.

1 $$\begin{aligned} |\theta _1 \rangle = H \bigotimes H^{\bigotimes n} | 1 \rangle \bigotimes ^1_{i=n} |x^0_i \rangle = \frac{1}{\sqrt{2^n}} \sum ^{2^n-1}_{x=0} |-\rangle |x\rangle \end{aligned}$$

In the quantum state vector $|\theta _1\rangle$, state $|0\rangle$ is used to encode a dominating set candidate with no vertices, state $|1\rangle$ is used to encode a candidate with only the vertex $v_1$, state $|2\rangle$ encodes a candidate with the vertex $v_2$, and so on with state $|2^n -1\rangle$ encoding a possible dominating set with *n* vertices.

**Figure 4:**
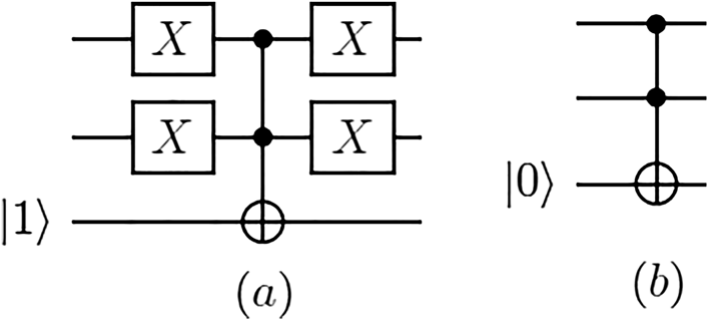
OR (**a**) and AND (**b**) operations of two Boolean variables.

### Finding legal dominating sets

The next step in the dominating set problem is to find all valid dominating sets among all the $2^n$ candidates. If a subset $U \subseteq V$ of vertices is an element of the $2^n$-large candidate space and at the same time *U* also satisfies that for all $v \in V-U$ there is a $u \in U$ for which $(u, v) \in E$, then this subset is a legal dominating set. Otherwise, it is not a legal dominating set. The dominating set problem consists in finding a legal dominating set with the minimum number of vertices. To that end, suppose that the *k*-th edge, $e_k = (v_i, v_j) \in G$ where $1 \le k \le z$ and that bits $x_i$ and $x_j$ are used to encode vertices $v_i$ and $v_j$, respectively. Assume further that the number of vertices adjacent to $v_i$ is $y_i$ where $1 \le i, y_i \le n$. Let $v_j (1 \le j \le n)$ be one of the adjacent vertices of $v_i$. By logic convention, we use $\vee$ to represent the logic OR operation. Then, a legal dominating set for any graph *G* can be regarded as a candidate among the $2^n$ possible candidates that satisfies each formula of the form $v_i \vee v_j$ where $v_j$ are vertices adjacent to $v_i$.

**Figure 5:**
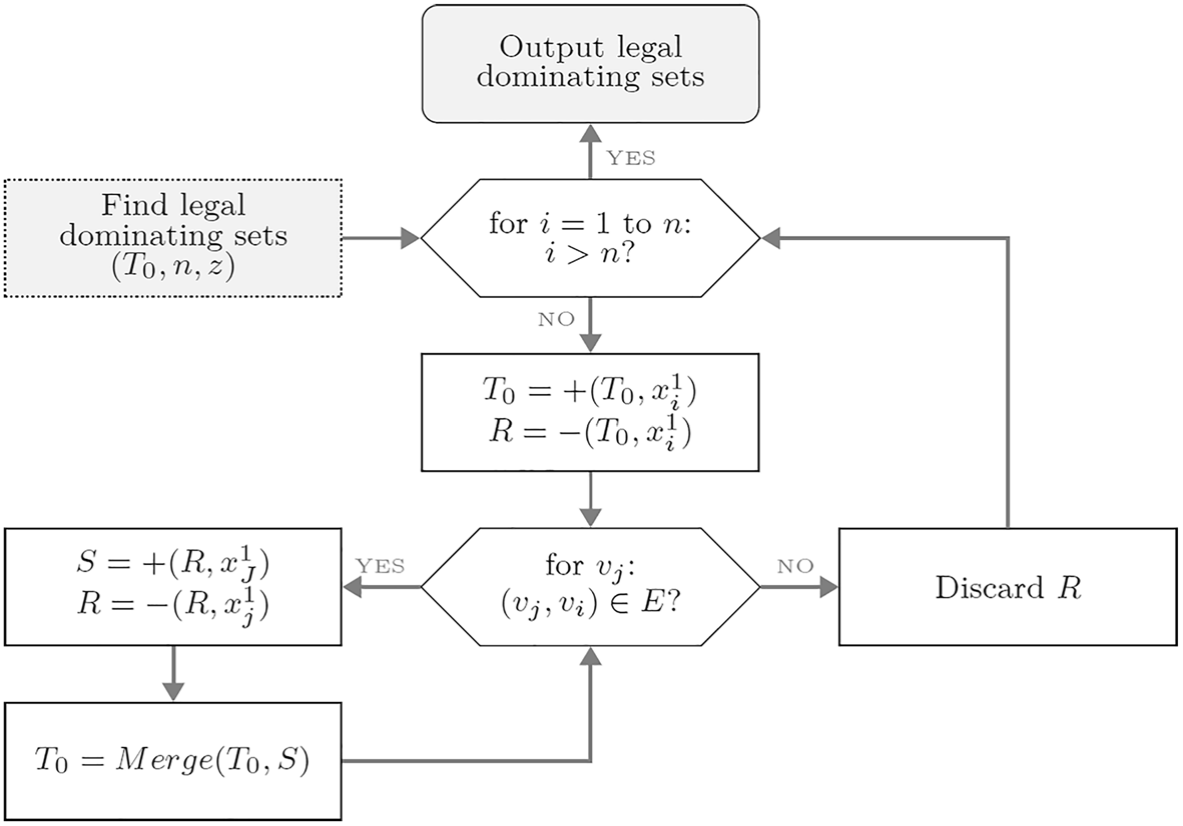
Biomolecular procedure for finding legal dominating sets.

The biomolecular procedure is given in Fig. 5. The *Extract* operation preserves legal dominating sets while removing illegal ones. In each step, tube $T_0$ will contain all of the strands with $x_i = 1$, while tube *R* will contain all of the strands with $x_i = 0$. Hence, from the definition of a dominating set, $T_0$ encodes *U* where vertex $v_i \in U$ and $v_i \notin V-U$, while *R* encodes *U* where $v_i \notin U$ and $v_i \in V-U$. If there are no other vertices adjacent to $v_j$ , then the *Discard* operation will remove the content of *R*, i.e. the illegal dominating sets in *R*. At the time the *Extract* operation is called on *S* and *R*, tube *S* consists of all the strands with $v_i = 0$ and $v_j = 1$, while tube *R* consists of all of the strands with $v_i=v_j=0$. Therefore, *S* contains the strands that encode legal dominating sets. Next, the content of *S* is poured into $T_0$. After executing the inner loop for all adjacent vertices $v_j$, $T_0$ contains the strands that satisfy $(v_i, v_j) \in E$, where $v_i \in U$ and $v_j \in V - U$, and *R* contains the strands that do not satisfy $(v_i, v_j) \in E$. At the end of the procedure, the remaining strands in $T_0$ encode legal dominating sets.

In the quantum case, the operations with which all legal dominating sets can be found are as given in (2) and (3). *I* is the identity matrix, $|r_{i,j} \rangle$ are *n* auxiliary quantum registers with *i*th register being of length $y_i + 1$, where $y_i$ the degree of a vertex $v_i$, while $|c\rangle$ is an auxiliary quantum register of $n+1$ qubits. The auxiliary registers $|r_{i,j}\rangle$ store the result of application of a logic OR operation to compute each clause of the form $x_i \vee x_j$, where $x_i$ ($x_j$) corresponds to vertex $v_i$ ($v_j$). The auxiliary register $|c\rangle$ stores the result of computing logic AND on those disjunctive clauses. Logic OR and AND can be implemented using the Toffoli operator as shown in Fig. 4. Despite having a rather complex look, formulas (2) and (3) are quite simple in nature and can be readily followed on the example circuit given in Fig. 5.

2 $$\begin{aligned}{} & {} |\theta _2 \rangle = \bigotimes ^1_{i=n+1} I \bigotimes ^1_{i=n} (\bigotimes ^1_{j=y_i} OR) \bigotimes I [|\theta _1 \rangle \bigotimes ^1_{i=n} \bigotimes ^1_{j=y_i} |r^1_{i,j} \rangle |r^0_{i,0} \rangle ] = \sum ^{2^n-1}_{x=0} | - \rangle | x \rangle \bigotimes ^1_{i=n} \bigotimes ^1_{j=y_i} | r_{i,j} \rangle |r^0_{i,0} \rangle \end{aligned}$$

3 $$\begin{aligned}{} & {} |\theta _3 \rangle = \bigotimes ^1_{i=n+1} I \bigotimes ^1_{i=n} (\bigotimes ^1_{j=y_i} I) \bigotimes I \bigotimes ^1_{i=n} AND \bigotimes I [|\theta _2 \rangle \bigotimes ^1_{i=n} |c^0_i \rangle |c^0_1 \rangle {]}\nonumber \\{} & {} \quad = \sum ^{2^n-1}_{x=0} | - \rangle | x \rangle \bigotimes ^1_{i=n} \bigotimes ^1_{j=y_i} | r_{i,j} \rangle |r^0_{i,0} \rangle \bigotimes ^1_{i=n} | c_i \rangle | c^1_0 \rangle \end{aligned}$$

### Finding minimum dominating sets

**Figure 6:**
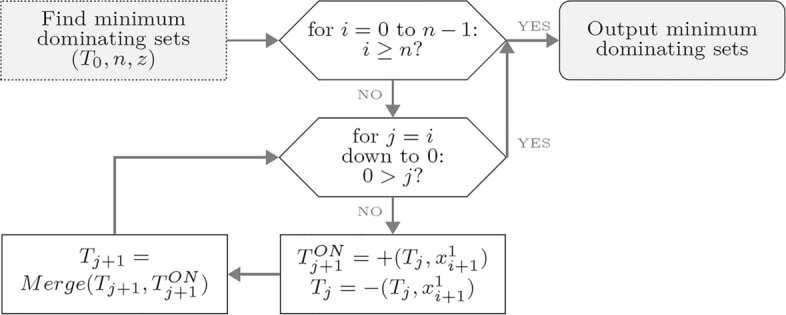
Biomolecular procedure for finding minimum dominating sets.

The biomolecular procedure for finding the minimum dominating sets from the set of legal dominating sets is given in Fig. 6. The procedure considers each vertex in a given graph (outer loop). At iteration (0, 0) the influence of vertex $v_1$, encoded as $x_1$, on the number of 1s in tubes $T_0$ and $T_1$ is computed. The *Extract* operation forms two different tubes, $T^{ON}_1$ and $T_0$ from $T_0$. Hence, $T^{ON}_1$ has $x_1 = 1$ with vertex $v_1$, while $T_0$ has $x_1 = 0$ without vertex $v_1$. In short, in iteration (0,0) a single 1 is recorded in $T^{ON}_1$ and no 1s are recorded in $T_0$. Next, in the *Merge* operation the content of $T^{ON}_1$ is poured into $T_1$. With this, the influence of vertex $v_1$ on the number of 1s equals a single 1 in $T_1$. Along these lines, the influence of all other vertices is calculated with each result *i* stored in tube $T_i$. Therefore, the procedure classifies each legal dominating set according to the number of 1s.

The quantum case requires further auxiliary qubits to implement the *Extract* and *Merge* operations in the biomolecular procedure that determines minimum dominating sets. These are qubits $|z_{i+1,j} \rangle$ and $|z_{i+1,j+1} \rangle$ for each $0 \le i \le n -1$ and $0 \le j \le i$. Each qubit $|z_{i+1,j} \rangle$ and $|z_{i+1,j+1} \rangle$ is initially prepared in state $|0 \rangle$. Following closely the biomolecular procedure, the first iteration, (0,0), will require a different treatment from all the other iterations. In the first iteration *i* and *j* are both set to zero and the implemented logic formulas are $|x_1 \rangle \wedge | c_n \rangle$ and $|\overline{x}_1 \rangle \wedge | c_n \rangle$, where $\wedge$ stands for the logic AND operation. These two operations are implemented together by the gate sequence: *CCNOT* with $|x_1 \rangle$ and $|c_n \rangle$ as controls and $|z_{1,1} \rangle$ as target; *NOT* on $|x_1 \rangle$; *CCNOT* with qubits $|\overline{x}_1 \rangle$ and $|c_n \rangle$ as controls and $|z_{1,0} \rangle$ as target; *NOT* on $|\overline{x}_1 \rangle$. With this, $|z_{1,0} \rangle$ will store the information that the number of 1s is 0, while $|z_{1,1} \rangle$ will store the information that the number of 1s is 1. All other iterations except the (0,0) are executed on $|x\rangle$ and $|z\rangle$ registers only. The general formula for iteration (*i*, *j*), i.e. for the computation of $|x_{i+1} \rangle \wedge | z_{i,j} \rangle$ and $|\overline{x_{i+1}} \rangle \wedge | z_{i,j} \rangle$ is: *CCNOT* with $|x_{i+1} \rangle$ and $|z_{i,j} \rangle$ as controls and $|z_{i+1,j+1} \rangle$ as target; *NOT* on $|x_{i+1} \rangle$; *CCNOT* with controls $|\overline{x_{i+1}} \rangle$ and $|z_{i,j} \rangle$ and target $|z_{i+1,j} \rangle$; *NOT* on $|\overline{x_{i+1}} \rangle$. After the contribution of vertex $v_{i+1}$ encoded by $x_{i+1}$ to the number of 1s is computed in the loop iteration (*i*, *j*) in the biomolecular procedure, $|z_{i+1,j+1} \rangle$ will record the information as to whether $T_{i+1}$ has (*j* + 1) 1s, and $|z_{i+1,j} \rangle$ will record the information as to whether a tube $T_j$ has *j* 1s. After completing the procedure the quantum state is given in (4).

4 $$\begin{aligned} |\theta _4 \rangle = |\theta _3 \rangle \bigotimes ^1_{i=n} \bigotimes ^0_{j=1} |z_{i,j} \rangle \end{aligned}$$

As the described procedure is quite complex, we provide a proof of its correctness in the following Lemma.

#### Lemma 0.1

**Figure 7:**
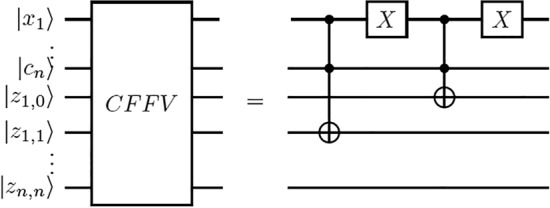
Quantum circuit for the CFFV routine.

**Figure 8:**
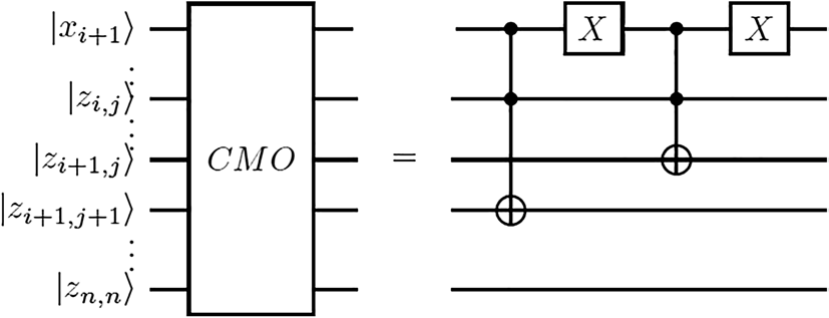
Quantum circuit for the CMO routine.

The Boolean circuit generated from calculating $T_{j+1}^{ON}$, $T_j$ and $T_{j+1}$ at the iteration (0, 0) in the molecular algorithm for finding minimum sized dominating sets is $|c_n\rangle \wedge |x_1\rangle$ and $|c_n\rangle \wedge |\overline{x_1}\rangle$. It can be implemented by the quantum circuit CFFV in Fig. 7. The Boolean circuit generated from the same calculations at the iteration (i, j) is $|x_{i+1}\rangle \wedge |z_{i,j}\rangle$ and $|\overline{x_{i+1}}\rangle \wedge |z_{i,j}\rangle$ and it can be implemented by the quantum circuit CMO in Fig. 8.

#### Proof

This proof is by induction. The Boolean circuit generated by implementing $T_{j+1}^{ON}$, $T_j$ and $T_{j+1}$ at the iteration (0, 0) in the algorithm for finding minimum sized dominating sets consists of solutions (legal dominating sets) in tube $T_1$ including the first vertex satisfying $|c_n\rangle \wedge |x_1\rangle$ and the solutions in tube $T_0$ not including the first vertex satisfying $|c_n\rangle \wedge |\overline{x_1}\rangle$. After the influence of vertex $v_1$ encoded by $x_1$ on the number of 1s is determined in the iteration (0, 0), $|z_{1,1}\rangle$ will record the status of tube (set) $T_1$ that has one 1 and $|z_{1,0}\rangle$ will record the status of tube (set) $T_0$ that has zero 1s.

Therefore, one CCNOT gate $| z_{1,1}^0 \oplus c_n \cdot x_1 \rangle$ is applied to implement $|c_n\rangle \wedge |x_1\rangle$ and one NOT gate is used on $x_1$ $(\overline{x_1})$ and another CCNOT gate $| z_{1,0}^0 \oplus c_n \cdot \overline{x_1} \rangle$ is used to implement $|c_n\rangle \wedge |\overline{x_1}\rangle$. Next, another NOT gate is applied to $\overline{x_1}$ $(|x_1\rangle )$ to restore $x_1$ in $|x_n\cdots x_1\rangle$ to its superposition state. This is to say that if $|z_{1,1}\rangle = |1\rangle$, then $|z_{1,1}^1\rangle$ is applied to indicate that the legal dominating sets in tube $T_1$ include the first vertex and have one 1, and if $|z_{1,0}\rangle = |1\rangle$, then $|z_{1,0}^1\rangle$ is used to indicate that the legal dominating sets in tube $T_0$ do not contain the first vertex and have no 1s. Therefore, the quantum circuit CFFV calculates $|c_n\rangle \wedge |x_1\rangle$ and $|c_n\rangle \wedge |\overline{x_1}\rangle$.

The Boolean circuit generated by implementing $T_{j+1}^{ON}$, $T_j$ and $T_{j+1}$ at the iteration (1, 1) includes legal dominating sets in tube $T_2$ consisting of the second vertex satisfying $|x_2\rangle \wedge |z_{1,1}\rangle$ and legal dominating sets in tube $T_1$ not containing the second vertex satisfying $|\overline{x_2}\rangle \wedge |z_{1,1}\rangle$. After the influence of $x_2$ on the number of 1s is determined at the iteration (1, 1), $|z_{2,2}\rangle$ records the status of tube $T_2$ that has two 1s and $|z_{2,1}\rangle$ records the status of tube $T_1$ that has one 1. Therefore, a CCNOT gate $|z_{2,2}^0 \oplus x_2 \cdot z_{1,1}\rangle$ implements $|x_2\rangle \wedge |z_{1,1}\rangle$ and a NOT gate on $x_2$ ($|\overline{x_2}\rangle$) and a CCNOT gate $|z_{2,1}^0 \oplus \overline{x_2} \cdot z_{1,1}\rangle$ are applied to implement $|\overline{x_2}\rangle \wedge |z_{1,1}\rangle$. Next, another NOT gate restores $\overline{x_2}$ $(|x_2\rangle )$ in $|x_n \cdots x_1\rangle$ to its superposition state. This indicates that if $|z_{2,2}\rangle =|1\rangle$, then $|z_{2,2}^1\rangle$ indicates that the legal dominating sets in tube $T_2$ include the second vertex and have two 1s, and if $|z_{2,1}\rangle =|1\rangle$, then $|z_{2,1}^1\rangle$ indicates that the legal dominating sets in tube $T_1$ do not consist of the second vertex and have one 1.

Next, when $i=j=t$ the Boolean circuit obtained by implementing $T_{j+1}^{ON}$, $T_j$ and $T_{j+1}$ at the iteration (t, t) is $|x_{t+1}\rangle \wedge |z_{t,t}\rangle$ and $|\overline{x_{t+1}}\rangle \wedge |z_{t,t}\rangle$. It can be implemented by two CCNOT gates $| z_{t+1,t+1}^0 \oplus x_{t+1} \cdot z_{t,t} \rangle$ and $| z_{t+1,t}^0 \oplus \overline{x_{t+1}} \cdot z_{t,t} \rangle$. Next, when $i=t$ and $j=t-1$, the circuit generated from calculating $T_{j+1}^{ON}$, $T_j$ and $T_{j+1}$ at the iteration (t, t-1) contains legal dominating sets in tube $T_{(t-1)+1}$ containing the $(t+1)$-th vertex satisfying $|x_{t+1}\rangle \wedge |z_{t,t-1}\rangle$ and the legal dominating sets in tube $T_{t-1}$ not including the $(t+1)$-th vertex satisfying $|\overline{x_{t+1}}\rangle \wedge |z_{t,t-1}\rangle$. After the influence of $x_{t+1}$ on the number of ones is established at the iteration (t, t-1), $|z_{t+1,(t-1)+1}\rangle$ records the status of tube $T_{(t-1) +1}$ that has *t* 1s and $|z_{t+1,t-1}\rangle$ records the status of tube $T_{t-1}$ that has $t-1$ 1s. Hence, a CCNOT gate $|z_{t+1,(t-1)+1}^0 \oplus x_{t+1} \cdot z_{t,t-1}\rangle$ is used to implement $|x_{t+1}\rangle \wedge |z_{t,t-1}\rangle$ and a NOT gate on $x_{t+1}$ $(|\overline{x_{t+1}}\rangle )$ and a CCNOT gate $|z_{t+1,t-1}^0 \oplus \overline{x_{t+1}} \cdot z_{t,t-1}\rangle$ are used to implement $|x_{t+1}\rangle \wedge |z_{t,t-1}\rangle$. Next, another NOT gate is applied to $\overline{x_{t+1}}$ $(|x_{t+1}\rangle )$ to restore $x_{t+1}$ in $|x_n \cdots x_1\rangle$ to its superposition state. This is to say that if $|z_{t+1,(t-1)+1}\rangle =|1\rangle$, then $|z_{t+1,(t-1)+1}^1\rangle$ will indicate that the legal dominating sets in tube $T_{(t-1)+1}$ contain the $(t+1)$-th vertex and have *t* 1s, and if $|z_{t+1,t-1}\rangle =|1\rangle$, then $|z_{t+1,t-1}^1\rangle$ indicates that the legal dominating sets in tube $T_{t-1}$ do not contain the $(t+1)$-th vertex and have $t-1$ 1s. Therefore, the quantum circuit CMO calculates $|x_{i+1}\rangle \wedge |z_{i,j}\rangle$ and $|\overline{x_{i+1}}\rangle \wedge |z_{i,j}\rangle$.

With the above, it is at once inferred that the Boolean circuit generated for implementing $T_{j+1}^{ON}$, $T_j$ and $T_{j+1}$ at the iteration (0, 0) calculates $|c_m\rangle \wedge |x_1\rangle$ and $|c_m\rangle \wedge |\overline{x_1}\rangle$ and it can be implemented by the quantum circuit CFFV. Likewise, the circuit obtained for implementing $T_{j+1}^{ON}$, $T_j$ and $T_{j+1}$ at the iteration (i, j) calculates $|x_{i+1}\rangle \wedge |z_{i,j}\rangle$ and $|\overline{x_{i+1}}\rangle \wedge |z_{i,j}\rangle$ and it can be implemented by the quantum circuit CMO. $\square$

### Reading out minimum dominating sets

Having found all the minimum dominating sets for a given graph, they need to be output. After completing the procedure that finds minimum dominating sets, the molecular solutions that contain the corresponding strands are located in tubes $T_0, T_1, \ldots , T_n$, respectively. The detection of answers is straight forward and involves introduction of DNA material that will bind with the solutions. This corresponds to the following procedure:

#### Procedure 0.2

Procedure readout($$T_0, \ldots , T_n, n$$)

For k = 1 to n:

      If (Detect ($T_k$) == “yes”) then

            Read ($T_k$) and terminate the algorithm.

      EndIf

EndFor

EndProcedure

In quantum terms, the process is more involved. At this point, the minimum dominating sets are marked as such but performing a measurement may not give the desired outcome. This is because these sets are marked by changing global phases but the probability of measuring them may be exponentially small at $1/2^n$ . In order to improve the probability of obtaining the right answer, we employ the routine of amplitude amplification. This routine constitutes an integral part of Grover’s algorithm and the reader is advised to consult Grover’s work^23^. The probability of success in measuring the minimum dominating set using amplitude amplification is at least 0.5.

**Figure 9:**
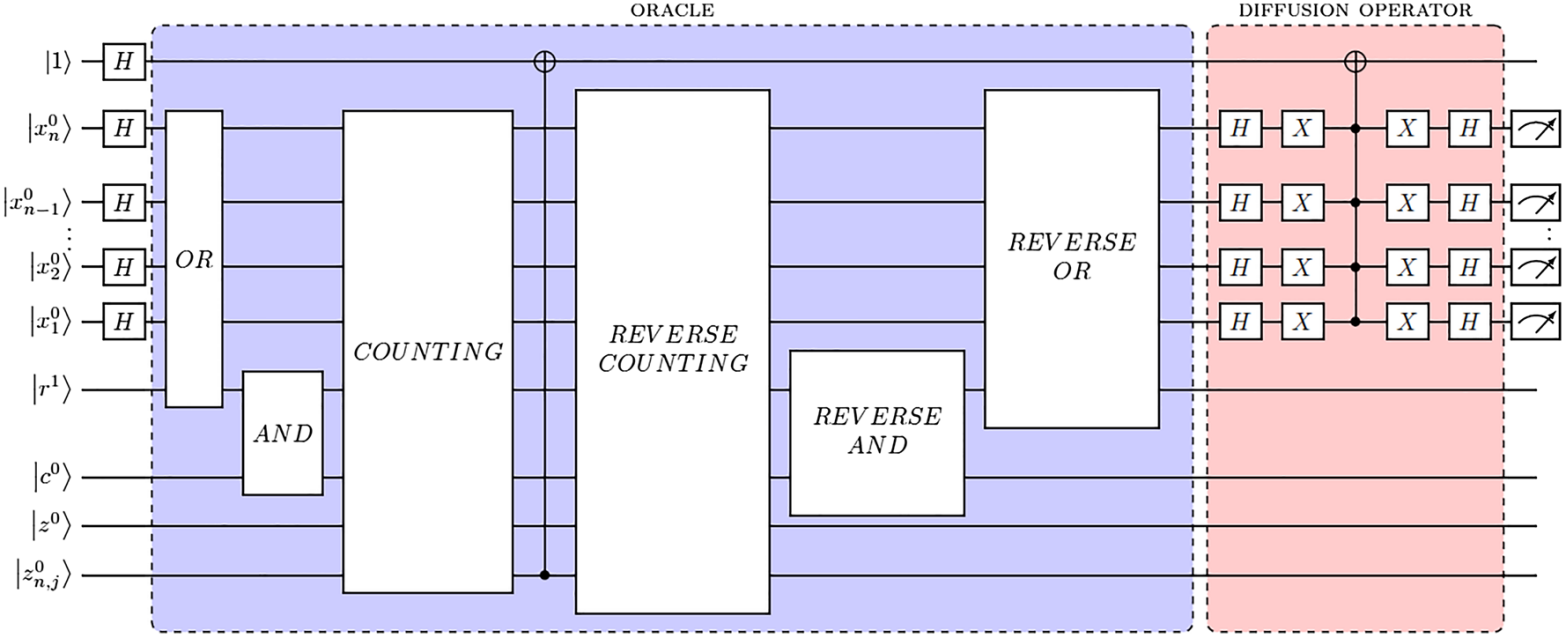
Circuit schematic for the entire quantum algorithm.

### Summary of the algorithm

Figure 9 shows the schematic of the quantum circuit for the entire algorithm. Except for the initial superposition state $|\theta _1 \rangle$ obtained through the Hadamard gates on the ancilla and the input qubits, the algorithm can be seen in terms of Grover’s search routine consisting of an oracle and a diffusion operation. The OR block consists of OR gates applied to the input state with ancillary qubit register $|r \rangle$ storing the outcome of each OR evaluation. Next, the AND block consists of AND gates applied to the $|r \rangle$ register and the $|c \rangle$ register in order to determine which subsets of a given graph are valid dominating sets. After the OR and AND blocks, the quantum system is in the state $|\theta _3 \rangle$. The block named COUNTING sums up the number of vertices for each dominating set in $|\theta _3 \rangle$ and stores the result in the auxiliary register $|z \rangle$. Then, the CNOT gate flips the phase of the ancillary qubit $| - \rangle$ for the dominating sets with the lowest number of vertices. After that, the three blocks of the oracle are run in reverse order to bring the system back to its superposition state. And lastly, the diffusion operator is applied to amplify the amplitude of those dominating sets selected by the oracle.

In order to establish the minimum number of vertices in a dominating set, the oracle has to be prepared to match the number of vertices and the algorithm is run for each oracle matrix until the minimum number of vertices is found. This overhead is linear in the input size *n*, which is the number of vertices of graph *G*.

## Mathematical representation of molecular solutions

In Chang and Guo^10^, a sticker (which is a specific DNA sequence) was used to encode each bit. The sticker-based model of computation was first proposed by Roweis et al.^24^ as an enhancement of the Adleman–Lipton model^16^. In our paper, we allow for the dominating set candidate space to be constructed in arbitrary way, without being restricted to the sticker-based encoding.

The following lemma is used to demonstrate how molecular solutions can be represented in terms of a unit vector in a finite-dimensional Hilbert space.

### Lemma 0.3

For solving the dominating set problem for any graph *G* with *z* edges and *n* vertices, molecular solutions can be represented in terms of a unit vector in a finite-dimensional Hilbert space.

### Proof

From the biomolecular procedure in Fig. 3 for constructing the dominating set candidate space, the $2^n$ candidates encoded by $2^n$ DNA sequences are generated, and are encoded using *n* Hadamard gates operating on *n* initial quantum bits that produces the new quantum state vector $|\theta _1\rangle$ in Eq. (1). This implies that the $2^n$ candidates encoded by $2^n$ DNA sequences are represented in terms of a unit vector in a finite-dimensional Hilbert space.

Next, the biomolecular procedure in Fig. 5 uses biological operations to implement logic OR and AND gates for deciding legal and illegal dominating sets among the $2^n$ dominating set candidates. Because logic OR and AND gates can be implemented by the Toffoli operator as shown in Fig. 4, the same task is also realised by unitary operators in Eqs. (2) and (3). The new quantum state vector $|\theta _3\rangle$ in Eq. (3) stores the result of determining legal and illegal dominating sets. This indicates that both the legal and the illegal dominating sets among the $2^n$ candidates are still expressed as a unit vector in a finite-dimensional Hilbert space.

Next, the biomolecular procedure for finding the minimum dominating sets in Fig. 6 classifies the legal dominating sets among the $2^n$ candidates according to the number of vertices. In light of the proof procedure of Lemma 0.1, each operation in every iteration in the biomolecular procedure in Fig. 6 can be implemented by the quantum circuit CFFV in Fig. 7 and the quantum circuit CMO in Fig. 8. The new quantum state vector $|\theta _4\rangle$ in Eq. (4) stores the result to classify legal choices according to the number of vertices. This implies that the legal dominating sets are represented in terms of a unit vector in a finite-dimensional Hilbert space.

Next, the biomolecular procedure $readout(T_0, \ldots , T_n, n)$ reads out the answer encoded by DNA strands with the minimum number of vertices and the answer is also read out by a measurement after the Grover operator is used to increase the amplitude of the answer in the quantum case. Hence, the answer encoded by DNA sequences is also represented by a unit vector in a finite-dimensional Hilbert space. Therefore, it is at once derived that for solving the dominating set problem for any graph *G* with *z* edges and *n* vertices, molecular solutions are represented in terms of a unit vector in a finite-dimensional Hilbert space. $\square$

## Complexity assessment

The time complexity of the proposed quantum algorithm that solves the dominating set problem is $O(2^{n/2})$ quantum gates and one measurement in the best case. In the worst case it is $O(2^{n/2})$ quantum gates and *n* measurements. The space complexity for the best and the worst case is $O(n^2)$ qubits. These results are readily obtained from circuit analysis.

**Figure 10:**
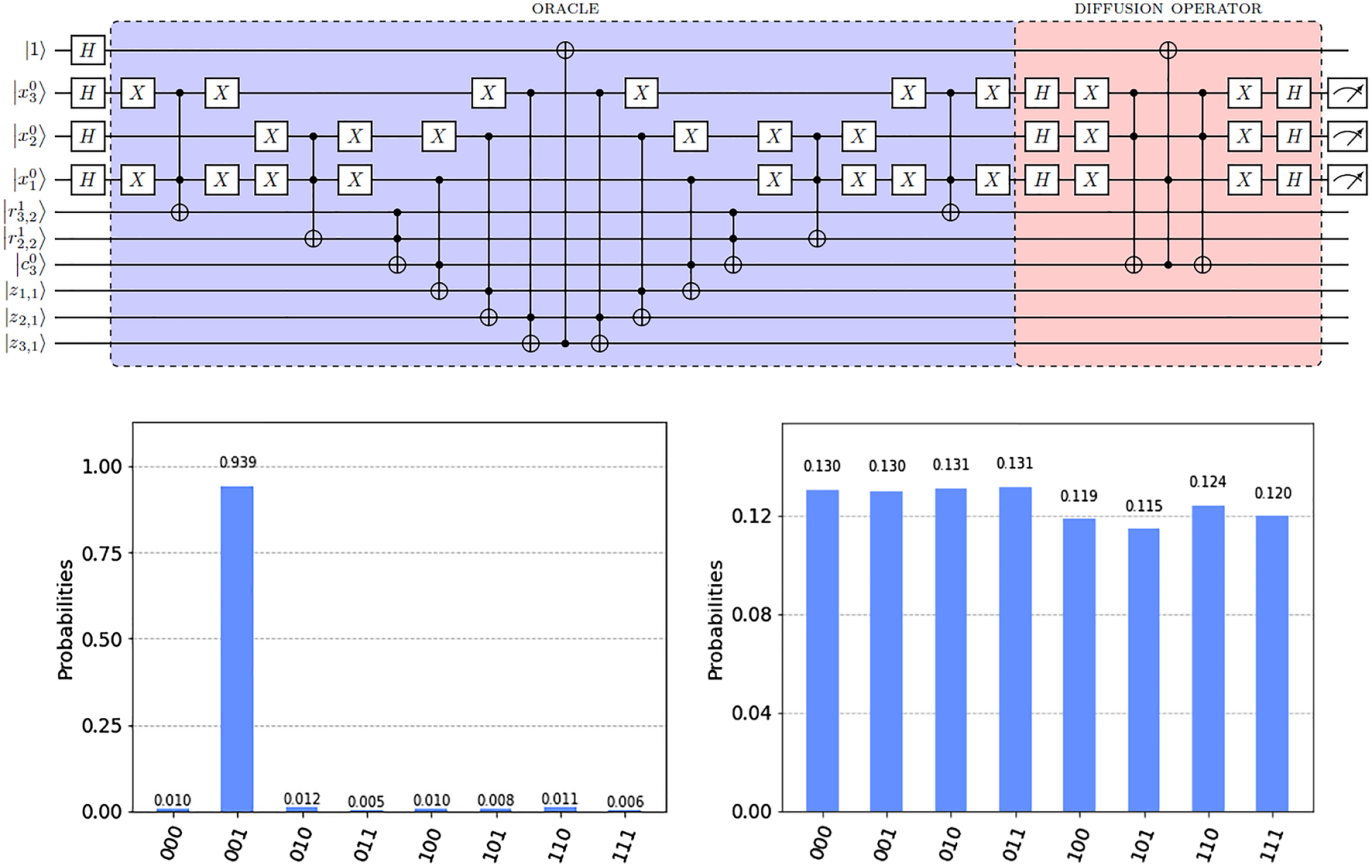
Above: Quantum circuit for a graph with three vertices and two edges in Fig. 1. The oracle and the diffusion operator are executed interchangeably two times in a row for optimal measurement output. Below: Result of executing the circuit on the IBM qasm simulator (left), and the Brooklyn backend (right).

## Experimental implementation

We implement the proposed quantum algorithm for a graph with three vertices and two edges and execute it on the IBM qasm simulator and the Brooklyn (a 65-qubit system) backend. Figure 10 shows a circuit for the graph, which is also given in Fig. 1. The graph in Fig. 1 has the following dominating sets: $\{v_1\}, \{v_1, v_2\}, \{v_1, v_3\}, \{v_1, v_2, v_3\}$, where only $\{v_1\}$ is minimal. The proposed algorithm finds this minimum set.

Figure 10 shows the output of an execution of the circuit on the qasm simulator and the Brooklyn backend. The dominating set $v_3 = 0, v_2 = 0, v_1 = 1$, is obtained with a high probability of 0.939 on the simulator. This is consistent with the theoretical probability of observing a single marked state after the amplitude amplification routine as described in^23^. On the other hand, the output from the backend shows a superposition state over all dominating set candidates. This result is expected and can be explained by considering the cumulative error caused by CNOT gates, which constitutes a major impact on circuit performance. The Brooklyn backend consists of 65 qubits and is based on IBM’s Hummingbird r2 processor. The average CNOT error rate for this backend is 1.255e$-$2 (as of March 1, 2022). Given that the circuit in Fig. 10 had to be decomposed into the native gate set for the backend in order to make it executable on the backend, at the point of execution it contained at least 182 CNOT gates. (Decomposition methods may vary depending on the processor and other factors. This is a theoretical number based on the fact that the currently most performance-effective decomposition of the Toffoli gate requires 6 CNOT gates^25^.) With this, the probability of obtaining the state 001 is only $(1\,-\,0.01255)^{182} \approx 1\%$. In this calculation we omitted all other possible sources of error, such as the error from single gates, the readout error and qubit decoherence times.

Despite quite strong limitations to what is possible to execute currently on state-of-the-art quantum devices, the number of available qubits and the fidelity of qubits keep increasing year by year. For instance, in November 2022, IBM released its newest 433-qubit quantum chip Osprey, thereby improving on its previously released Eagle chip of 127 qubits in 2021. By 2025, IBM hopes to release their 4158+ large quantum chip Kookaburra. With these improvements, the feasibility of executing our quantum algorithm on actual quantum devices also increases. We hope to be able to demonstrate our algorithm on real world examples in the not so distant future.

## Conclusion

Dominating sets are indispensable in any large-scale network organization and scheduling. Among their many applications, dominating sets play an important role in efficient allocation of resources and preserving the lifetime of sensor networks. In the present article, we have shown that the dominating-set problem for an arbitrary quantum network represented as a graph *G* with *n* vertices and *z* edges can be solved with O($2^{n/2}$) queries and O($n^2$) qubits using our proposed quantum algorithm. We have furthermore demonstrated that this quantum algorithm is the best known to date for the dominating-set problem, thereby confirming the theoretical results given in^4^. The correctness of the proposed quantum algorithm was confirmed by testing it on the example graph instance in Fig. 1 using IBM Quantum’s simulator and the Brooklyn backend, which is one of the largest state-of-the-art systems available today. This result makes a step forward for an efficient implementation of dominating sets in the near-term and future quantum communications and networking architectures.

## Data Availability

The datasets and code used and analysed during the current study are available from the corresponding author on reasonable request.
